# Methamphetamine-induced conditioned place preference in LG/J and SM/J mouse strains and an F45/F46 advanced intercross line

**DOI:** 10.3389/fgene.2012.00126

**Published:** 2012-07-11

**Authors:** Camron D. Bryant, Loren A. Kole, Michael A. Guido, Riyan Cheng, Abraham A. Palmer

**Affiliations:** ^1^Department of Human Genetics, The University of Chicago,Chicago, IL, USA; ^2^Department of Psychiatry and Behavioral Neuroscience, The University of Chicago,Chicago, IL, USA

**Keywords:** addiction, amphetamine, cue-associated craving, drug abuse, locomotion, pavlovian conditioning, psychostimulants, reinforcement

## Abstract

The conditioned place preference (CPP) test is frequently used to evaluate the rewarding properties of drugs of abuse in mice. Despite its widespread use in transgenic and knockout experiments, there are few forward genetic studies using CPP to identify novel genes contributing to drug reward. In this study, we tested LG/J and SM/J inbred strains and the parents/offspring of 10 families of an F_45_/F_46_ advanced intercross line (AIL) for methamphetamine-induced CPP (MA-CPP) once per week over 2 weeks. Both LG/J and SM/J mice exhibited significant MA-CPP that was not significantly different between the two strains. Furthermore, LG/J mice showed significantly less acute MA-induced locomotor activity as well as locomotor sensitization following subsequent MA injections. AIL mice (*N* = 105) segregating LG/J and SM/J alleles also demonstrated significant MA-CPP that was equal in magnitude between the first and second week of training. Importantly, MA-CPP in AIL mice did not correlate with drug-free or MA-induced locomotor activity, indicating that MA-CPP was not confounded by test session activity and implying that MA-CPP is genetically distinct from acute psychomotor sensitivity. We estimated the heritability of MA-CPP and locomotor phenotypes using midparent-offspring regression and maximum likelihood estimates derived from the kinship coefficients of the AIL pedigree. Heritability estimates of MA-CPP were low (0–0.21) and variable (SE = 0–0.33) which reflected our poor power to estimate heritability using only 10 midparent-offspring observations. In sum, we established a short-term protocol for MA-CPP in AIL mice that could reveal LG/J and SM/J alleles important for MA reward. The use of highly recombinant genetic populations like AIL should facilitate the identification of these genes and may have implications for understanding psychostimulant abuse in humans.

## INTRODUCTION

Vulnerability to psychostimulant abuse is heritable in humans and ranges from 0.4 to 0.7 (Goldman et al., 2005; Ho et al., 2010). Sensitivity to the subjective response to drugs of abuse is also heritable (Nurnberger et al., 1982; Crabbe et al., 1983) and can predict drug dependence (Haertzen et al., 1983; Schuckit and Smith, 2001; Fergusson et al., 2003). It is hypothesized that genetic variants affecting the subjective rewarding properties will sometimes be shared with those that affect drug abuse (Palmer and de Wit, 2012); indeed, there is some evidence to support this hypothesis (Ho et al., 2010; Hart et al., 2012).

We and others previously identified quantitative trait loci (QTL) influencing the acute locomotor response to methamphetamine (MA) in mice (Palmer et al., 2005; Phillips et al., 2008; Bryant et al., 2009, 2012b; Cheng et al., 2010; Parker et al., 2012). The locomotor stimulant and rewarding properties drugs of abuse are in part mediated by shared neurobiological mechanisms involving dopamine release in the nucleus accumbens (Di Chiara and Imperato, 1988). For this reason, locomotor activity may be regarded as a proxy for activation of the mesolimbic reward circuitry whose genetic basis may provide insight into the mechanisms governing the motivational properties of drugs of abuse in mice and humans (Wise and Bozarth, 1987). We previously used QTL, congenic, reverse genetic, and pharmacological analysis of the locomotor stimulant response to drugs of abuse in mice to identify casein kinase 1-epsilon as a genetic regulator of sensitivity to psychostimulants and opioids (Bryant et al., 2012b). This same gene in humans was associated with the euphoric properties of amphetamine (Veenstra-VanderWeele et al., 2006) and the addictive properties of heroin (Levran et al., 2008), supporting the rationale behind studying the locomotor stimulant response to drugs of abuse. However, the human candidate gene association studies have not yet been replicated.

While focusing on the genetic basis of the psychomotor properties of drugs of abuse has been fruitful, an alternative approach is to focus on a phenotype that is validated as a measure of the motivational properties of drugs of abuse in both mice and humans. In the conditioned place preference (CPP) test, subjects express their preference for one of two environments both before and after this environment has been paired with drug administration. Drug-induced CPP in rodents can predict whether a drug induces euphoria in humans (Tzschentke, 2007). Furthermore, humans also express CPP for a drug-paired environment that is highly correlated with the self-reported positive subjective effects of amphetamine (Childs and de Wit, 2009, 2011). This makes CPP an especially attractive phenotype for translational genetic studies, provided that it is a heritable trait.

We previously used a LG/J × SM/J F_34_ advanced intercross line (AIL) to map QTLs influencing MA-induced locomotor activity (Cheng et al., 2010). The LG/J strain and SM/J strains were originally selected for large and small body sizes from separate populations and were subsequently accessioned by The Jackson Laboratory where they were sib-sib mated to the fully inbred state (Cheverud et al., 1996). Breeding for AIL mice began in Dr. Jim Cheverud’s laboratory where unrelated individuals (non-sibs) were deliberately outcrossed for several generations. We obtained the AIL from Dr. Cheverud’s laboratory at generation F_33_ and this population has now progressed to generation F_50_, yielding a highly recombinant population that is ideal for fine mapping QTLs (Darvasi and Soller, 1995).

Our objectives in the present study were to determine whether or not LG/J and SM/J parental strains showed a significant difference in MA-induced CPP (MA-CPP; which would implicate a genetic basis) and to evaluate MA-CPP in AIL mice with the goal of using this population for mapping its genetic basis. We phenotyped the LG/J and SM/J parental inbred strains and the parents and offspring of 10 families of AIL mice. Next, we examined the correlation of MA-CPP with the locomotor activity measures exhibited during training and testing to determine whether this trait was distinct from MA psychomotor sensitivity and whether it might be influenced by variation in activity levels during preference assessment. We then calculated narrow-sense heritability estimates of MA-CPP and the locomotor phenotypes measured during preference assessment and CPP training. Narrow-sense heritability represents the proportion of phenotypic variance explained by additive genetic variance (Visscher et al., 2008); traits must be heritable for QTL mapping studies to succeed.

## MATERIALS AND METHODS

### MICE

All experiments were performed in accordance with the National Institutes of Health Guidelines for the Use of Laboratory Animals and were approved by the Institutional Animal Care and Use Committee at the University of Chicago. A 12 h/12 h light/dark cycle (lights on at 0600 hours) was used in the mouse colony room. Behavioral testing was conducted between 0800 and 1700 hours. Mice were same-sex housed in standard shoebox cages with corncob bedding in groups of 2–5 per cage. Thirty-one LG/J mice (13 females, 18 males) and 31 SM/J mice (15 females, 16 males) were tested for MA-CPP. The first of this cohort was purchased from The Jackson Laboratory (Bar Harbor, ME, USA) and the second cohort was subsequently bred in house.

LG × SM F_45_/F_46_ AIL mice were originally obtained from Jim Cheverud’s laboratory at Washington University in St Louis at the F_33_ generation and have since been bred and maintained in our laboratory by breeding 50–70 families per generation in which breeding pairs are chosen in a systematic manner in order to minimize relatedness. We used 105 AIL mice (55 females, 50 males) for MA-CPP. Twenty of these individuals (10 females, 10 males) were from the F_45_ generation and each of these individuals was from a different F_44_ family. These mice were phenotyped for MA-CPP and then paired randomly to form 10 families (10 dams, 10 sires). Mice were not selected based on their phenotypes. Eighty-five F_46_ offspring were generated from these 10 families (*N* = 4–11 per family) and thus, many of these offspring were siblings. An additional 32 F_44_ AIL mice (*N* = 16 females, 16 males) from four different F_43_ families were used in a saline (SAL) control experiment where both sides of the CPP apparatus were paired with SAL during training. All mice were 7–12 weeks at the beginning of testing.

### CONDITIONED PLACE PREFERENCE

We divided the open field boxes (37.5 cm × 37.5 cm; AccuScan Instruments, Columbus, OH, USA) into two equally sized, distinct sides using a 30-cm tall plastic black divider with a 5 cm × 5 cm mouse entryway excised from the bottom, middle part of the divider. The other three walls of each side were distinguished by visual cues (stripes on the walls) and tactile cues (floor textures; Bryant et al., 2012a). For confinement during MA and SAL trials, we turned the divider upside down so that the entryway was not accessible.

Mice were always administered MA (2 mg/kg i.p.) on the left side of the apparatus (white horizontal stripes, smooth floor texture) and SAL (i.p.) on the right side (black vertical stripes, pointed floor texture). The left side was, on average, the slightly less preferred side – there was approximately a 2–6% less initial preference for the drug-paired side in LG/J, SM/J, and AIL mice (observed in pilot studies and in the current study). We purposefully chose not to use a counterbalanced design in order to avoid interactions of drug treatment and/or genotype with a particular environment; instead, drug administration was always paired with the left side.

**FIGURE 1:**
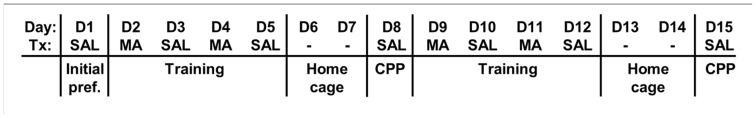
**Schematic of the MA-CPP protocol**. We used a 15-day protocol whereby mice were assessed for initial preference for the drug-paired side (D1, initial preference), trained for two separate 4-day sessions with alternating injections of MA and SAL in the respective contexts (D2–D5; D9–D12) and assessed for MA-CPP on Day 8 (D8) and Day 15 (D15). Each day represents a 30 min session. Day, day of the protocol; Tx, treatment for that day; SAL, saline treatment; MA, methamphetamine treatment; -, mice were left undisturbed in their home cage in the vivarium on these days.

A schematic of the MA-CPP protocol is shown in **Figure 1;** all test sessions and training sessions lasted 30 min. On the first day [Day 1 (D1)], mice were assessed for initial preference for the two sides of the apparatus. Following a SAL injection (10 ml/kg, i.p.), mice were placed into the SAL-paired side facing the open entryway to the drug-paired side. The time spent on the drug-paired side and the total distance traveled were recorded using the automated Versamax CPP and activity programs. Next, two series of four conditioning trials were performed in which mice received either 2 mg/kg MA (i.p.; D2, D4, D9, and D11) or SAL (i.p.; D3, D5, D10, and D12) and were confined to the MA- or SAL-paired side; in this manner one side was always associated with MA and the other side with SAL. After each session, mice were placed back in their home cages. The mice were left undisturbed on D6–D7 and D13–D14. We examined MA-CPP on D8 following the first series of training trials and on D15 following the second series of training trials whereby mice were administered a SAL injection (10 ml/kg, i.p.), placed into the saline-paired side facing the entryway, and provided open access to both sides and assessed for time spent on the drug-paired side. Thus, on D1, D8, and D15, we obtained a measure of preference for the drug-paired side and on D8 and D15 this increase in preference was interpreted as a conditioned reward resulting from an association of the effects of the drug with the drug-paired side.

### BEHAVIORAL ANALYSIS

After establishing that sex did not interact with strain, we combined sexes for all analyses. MA-CPP was analyzed using repeated measures ANOVA (D1, D8, and D15) and/or paired *t*-test of the time spent on the drug-paired side on D1, D8, and D15. The cut-off for significance of the comparisons between strains and between days was Bonferroni-corrected for multiple comparisons (0.05/number of comparisons; see Section “Results” for specific alpha levels). Locomotor activity in the LG/J and SM/J parental strains was analyzed using repeated measures ANOVA separately for the MA training days (D2, D4, D9, and D11) and SAL training days (D3, D4, D10, and D12) followed by unpaired *t*-tests to determine the source of strain effect and paired *t*-test to determine the source of strain × time interaction (Bonferroni-corrected). As an additional measure of MA-CPP in AIL mice, we calculated the difference in time spent on the MA-paired side between D1 and D8 (D8–D1) and examined its correlation among variables as well as estimated its heritability.

### HERITABILITY ESTIMATES

We estimated the narrow-sense heritability (*h*^2^), or the proportion of phenotypic variance explained by additive variance, using two methods. First, we used midparent-offspring linear regression of the F_45_ and F_46_ generations of AIL mice whereby *h*^2^ equals the slope of the regression line of the offspring averages of the 10 families regressed onto the midparent averages; the standard error of the estimate (SE) represents the standard error of the regression coefficient. Second, we used a mixed model that accounted for genetic relatedness among individuals. Here, we used the complete AIL pedigree (F_1_–F_47_) to calculate the kinship coefficients and partitioned the variance into genetic and non-genetic components, and then estimated the heritability using the maximum likelihood estimates of the variance components (Abney et al., 2000). The standard error of the heritability estimate was calculated using the jackknife resampling method (Shao and Wu, 1989) from 50 different subsets of the original data with ten observations being deleted.

## RESULTS

### MA-CPP IN LG/J AND SM/J STRAINS

**FIGURE 2:**
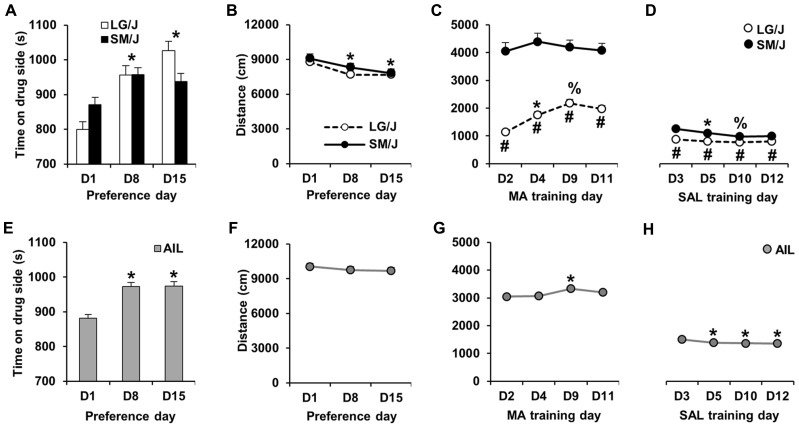
**MA-CPP and MA-ACT in LG/J, SM/J, and AIL mice. (A,E)** Time spent on the MA-paired side (s) on D1 and then on D8 and D15 following alternating MA and SAL trials. LG/J mice (*N* = 31) are represented by white bars, SM/J mice (*N* = 31) are represented by the black bars, and AIL mice (*N* = 105) are represented by gray bars. **(B,F)** Locomotor activity (total distance traveled in cm; summed over 30 min) in LG/J (white circles), SM/J (black circles), and AIL mice (gray circles; *N* = 105) during days of preference assessment. **(C,G)** MA-induced locomotor activity during MA training trials (D2, D4, D9, and D11) in LG/J, SM/J, and AIL mice. **(D,H)** Locomotor activity during SAL training trials (D3, D5, D10, and D12) in LG/J, SM/J, and AIL mice. *Significantly different from D1 **(A,B,E)**, D2 **(C,G)**, or D3 **(D,H)**. ^#^Significantly different from SM/J **(C,D)**. ^%^Significantly different from D4 **(B)** or D5 **(C)**. All significant results were Bonferroni-corrected for multiple comparisons across days (see Results).

A schematic of the MA-CPP protocol is shown in **Figure 1**. Following the assessment of initial preference on D1, MA-CPP was assessed twice; once on D8 and again on D15. **Figure 2A** illustrates MA-CPP in LG/J (*N* = 31) and SM/J strains (*N* = 31). When treating D1, D8, and D15 as repeated measures, there was a main effect of day (*F*_2,120_ = 22.4; *p* < 0.05) but the strain × day interaction was not statistically significant (*F*_2,120_ = 2.83; *p* = 0.06). Therefore, the data were collapsed across strain and we observed a significant increase in time spent on the drug-paired side on D8 compared to D1 (*t*_61_ = 5.77; *p* < 0.017, Bonferroni-corrected; **Figure 2A)** and on D15 relative to D1 (*t*_61_ = 5.25; *p* < 0.017). There was no further increase in preference from D8 to D15 (*t*_61_ < 1), indicating maximal MA-CPP on D8 (**Figure 2A**).

### LOCOMOTOR ACTIVITY IN LG/J AND SM/J MICE

In examining locomotor activity during the days of preference assessment (D1, D8, and D15), there was no effect of strain (*F*_1,60_ < 1), an effect of day (*F*_2,120_ = 12.9; *p* < 0.0001), and no strain × day interaction (*F*_2,120_ < 1). The effect of day was explained by mice showing a significant decrease in locomotor activity on D8 and D15 relative to D1 (*t*_61_ = 3.47, 4.43; *p* < 0.017; Bonferroni-corrected; **Figure 2B**).

**Figure 2C** illustrates locomotor activity during MA training days (D2, D4, D9, and D11) in LG/J and SM/J mice. Repeated measures ANOVA of the four MA training days revealed a main effect of strain (*F*_1,60_ = 68.55; *p* < 0.05), day (*F*_3,180_ = 17.61; *p* < 0.05) and a strain × day interaction (*F*_3,180_ = 12.00; *p* < 0.05). The effect of strain was explained by LG/J mice showing significantly less activity than SM/J mice on all 4 days (*t*_60_ = 9.09, 7.88, 6.84, 7.21; *p* < 0.013, Bonferroni-corrected). The strain × time interaction was explained by LG/J showing a sensitized response to MA from D2 to D4 and a further sensitized response between D4 and D9 (*t*_30_ = 5.71, 4.44; *p* < 0.013). In contrast, SM/J mice did not show any significant increase in MA activity (MA-ACT) relative to D2 (*p* > 0.013; **Figure 2C**).

**Figure 2D** illustrates locomotor activity during SAL training days (D3, D5, D10, and D12). There was a significant main effect of strain (*F*_1,60_ = 21.58; *p* < 0.05) and a strain × time interaction (*F*_3,180_ = 4.21; *p* < 0.05). The effect of strain was explained by LG/J showing significantly less activity than SM/J mice on all 4 days (*t*_60_ = 4.80, 4.16, 2.93, 3.18, *p* < 0.013; Bonferroni-corrected). The interaction was explained by SM/J mice showing significant habituation from D3 to D5 and from D5 to D10 (*t*_30_ = 3.42, 3.35; *p* < 0.0083; Bonferroni-corrected). In contrast, LG/J mice did not show any significant decrease in activity following D3 (*p* > 0.0083; **Figure 2D)**

### MA-CPP AND LOCOMOTOR ACTIVITY IN AIL MICE

In examining MA-CPP in AIL mice (*N* = 105), paired *t*-test indicated a significant increase in preference for the MA-paired side on D8 and D15 relative to D1 (*t*_104_ = 5.89, 5.74; *p* < 0.017; Bonferroni-corrected), with no difference between D8 and D15 (*t*_104_ < 1; **Figure 2E**). Because of this last observation, we focused our correlations and heritability calculations on variables measured from D1 to D8 (see below).

In examining locomotor activity during the days of preference assessment (D1, D8, and D15), there was no significant change in activity on D8 or D15 relative to D1 (*t*_104_ = 1.3, 1.59; *p* > 0.017; Bonferroni-corrected; **Figure 2F**).

There was a small but significant increase in MA-induced locomotor activity in AIL mice on D9 relative to D2 (*t*_104_ = 2.83; *p* < 0.0083; Bonferroni-corrected; **Figure 2G**), which was the only indication of locomotor sensitization. For SAL treatment days, there was a significant decrease in locomotor activity on D5, D10, and D12 relative to D3 (*t*_104_ = 3.88, 3.78, 4.22; *p* < 0.0083), indicating a persistent locomotor habituation (**Figure 2H**).

### SAL CONTROL EXPERIMENT IN AIL MICE

**FIGURE 3:**
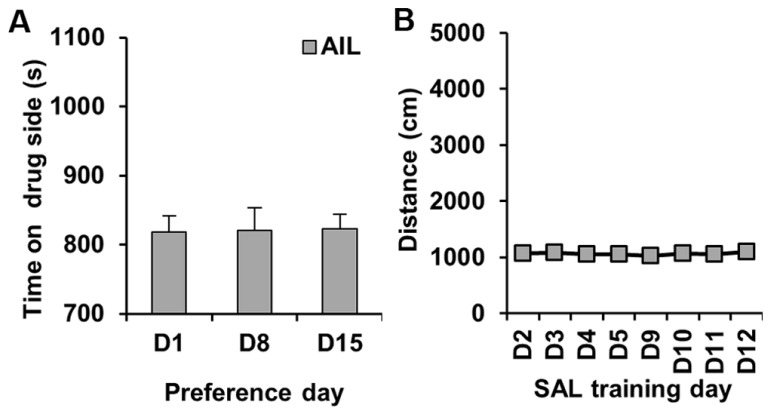
**Preference and locomotor activity in a SAL control experiment in AIL mice**. **(A)** Time spent on the drug (left)-paired side on D1 and on D8 and D15 following training with SAL administration in both sides of the CPP apparatus in AIL mice (*N* = 32). **(B)** Locomotor activity (centimeter) during the training trials where mice received SAL each day (D2–D5; D9–D12).

We performed a control experiment in which AIL mice received SAL on both sides; the purpose of this study was to confirm that we would not see any CPP under these conditions. As expected, there was no significant change in preference for either side on D8 or D15 relative to D1 (*t*_31_ < 1; **Figure 3A**). Similarly, there was no change in activity following SAL treatment on D3, D4, D5, D9, D10, or D11 relative to D2 (*t*_31_ < 1; **Figure 3B**).

### CORRELATION OF MA-CPP WITH D1 PREFERENCE AND LOCOMOTOR PHENOTYPES IN AIL MICE

Because AIL mice segregate alleles from both LG/J and SM/J strains, a correlation between two phenotypes implies that the two traits may be genetically correlated – that is, shared alleles contribute to both traits. Alternatively, a common environmental factor could drive such a correlation. We wanted to determine if MA-CPP was correlated with locomotor activity during training or preference assessment. Because AIL mice showed maximum MA-CPP on D8 (**Figure 2E**), we focused on phenotypes that were measured from D1 to D8 (**Table 1**).

**Table 1:** Correlation of MA-CPP with D1 preference and locomotor phenotypes in AIL mice.

Phenotype	D8 CPP (s)	D8-D1 CPP (s)
D1 preference (s)	0.05	-0.69*
D8 CPP (s)	1	1
D8-D1 CPP (s)	-0.69*	1
D1 ACT (cm)	0.13	0.03
D8 ACT (cm)	0.07	0.05
MA-ACT (cm)	0.05	-0.09
SAL-ACT(cm)	0.03	0.03

N = 105 (10 dams, 10 sires, 85 offspring). The variables include time spent on thedrug-paired side on D1 (D1 preference) and D8 (D8 CPP), difference in time spent on the drug-paired side (D8’D1 CPP), locomotor activity during preference assessment on D1 and D8 (D1 ACT and D8 ACT), and the average locomotor activity during MA trials (D2, D4; MA-ACT) and SAL training trials (D3, D5; SAL-ACT). *p < 0.05 for Pearson’s correlation coefficient..

Importantly, MA-CPP as measured by the time spent on the drug-paired side on D8 (D8 CPP) showed virtually zero correlation with time spent on the drug-paired side on D1 (D1 preference; *r* = 0.05; *p* > 0.05; **Table 1**), indicating that the final preference for the drug-paired side was not dependent on an initially low or high preference for it. MA-induced locomotor activity on D2 and D4 and SAL-induced locomotor activity on D3 and D5 were both highly correlated (*r* = 0.77, 0.71; *p* < 0.05; data not shown); therefore, we averaged across D2 and D4 for MA-ACT and across D3 and D5 for SAL activity (SAL-ACT) in comparing their relationship with the preference variables.

**FIGURE 4:**
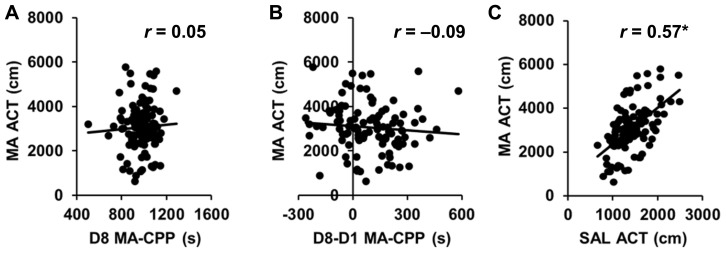
**No correlation of MA-CPP with MA-ACT in AIL mice**. **(A,B)** Scatterplot of the average amount of MA-induced locomotor activity on D2 and D4 of training (MA-ACT) versus the time spent on the drug-paired side on D8 (**A**; D8 MA-CPP) or versus the difference in time spent on the MA-paired side between D1 and D8 (**B**; D8-D1 MA-CPP) in AIL mice (*N* = 105). **(C)** Scatterplot of MA-ACT versus the average amount of SAL-induced locomotor activity during D3 and D5 of training (SAL-ACT) in AIL mice. *r*, Pearson’s correlation coefficient. *p < 0.05.

While D8 CPP and D8-D1 CPP were highly correlated (*r* = 0.69; *p* < 0.05), neither of these MA-CPP measures correlated with any measures of locomotor activity, including D1 ACT, D8 ACT, MA-ACT (**Table 1;** **Figures 4A,B**), or SAL-ACT (*r* = -0.09 to 0.13; *p* > 0.05; **Table 1**), indicating that MA-CPP is not influenced by test session activity and implicating that the alleles regulating the rewarding properties of MA are largely separate from those regulating MA-ACT. Last, as expected based on our previous observations with open field activity in AIL mice, MA-ACT and SAL-ACT were highly correlated (*r* = 0.57; *p* < 0.05; **Figure 4C**).

### HERITABILITY OF MA-CPP AND LOCOMOTOR PHENOTYPES IN AIL MICE

For the measures involving prior or present MA treatment (D8 CPP, D8-D1 CPP, MA-ACT), the heritability estimates were generally much lower than for locomotor activity following SAL treatment (D1 ACT, D8 ACT, SAL-ACT). Specifically, the heritability of D8 CPP ranged from 0 to 0.21, the heritability of D8-D1 CPP ranged from 0 to 0.12, the heritability of MA-ACT ranged from 0 to 0.1, and the heritability estimates for drug-free locomotor activity following SAL treatment ranged from 0.33 to 0.68 (**Table 2**). Interestingly, there was a large strain difference in MA-ACT in LG/J and SM/J mice (**Figure 2C**); thus, the low heritability of MA-ACT cannot be explained by a lack of polymorphisms that contribute to the trait. Conversely, there was no parental strain difference in drug-free test session activity on D1 and D8 (**Figure 2B**), yet the heritability of these two phenotypes was reliably moderate or high (**Table 2**).

**Table 2:** Heritability estimates of preference and locomotor phenotypes in AIL mice.

Phenotype	h2 (SE): midparent-offspring regression	h2 (SE): maximum likelihood
D1 preference (s)	0.17 (0.14)	0.00 (0.00)
D8 CPP (s)	0.21 (0.10)	0.00 (0.33)
D8-D1 CPP (s)	0.12 (0.10)	0.00 (0.00)
D1 ACT (cm)	0.34 (0.23)	0.45 (0.33)
D8 ACT (cm)	0.52 (0.21)	0.68 (0.17)
MA-ACT (cm)	0.1 (0.13)	0.00 (0.31)
SAL-ACT(cm)	0.33 (0.19)	0.62 (0.29)

*N = 105 (10 dams, 10 sires, 85 offspring). The first column lists the various preference and locomotor measures (ACT). The second column represents the heritability estimates (h2) using midparent-offspring regression of the F45 and F46 generations of AIL mice. The third column represents the heritability estimates obtained when accounting for genetic relatedness using maximum likelihood estimation. SE, standard error of the estimate.*.

## DISCUSSION

LG/J, SM/J, and AIL mice all exhibited MA-CPP and there was no statistically significant difference in MA-CPP between the LG/J and SM/J parental inbred strains (**Figure 2A**) – a strain difference would have suggested a genetic basis. However, the lack of strain difference may be explained by several factors other than a lack of genetic contribution. First, even though our sample size was large (*N* = 31 per strain), we may have been underpowered to detect a strain difference. In support, LG/J mice trended toward a greater increase in MA-CPP on D15 relative to D1 (**Figure 2A**) and this difference was significant when considering just the D1 versus D15 comparison (data not shown). Previous studies demonstrated that genetic variation contributes to motivational properties of MA in mice as indicated by a correlated response to selection for MA oral consumption and MA-CPP (Wheeler et al., 2009; Scibelli et al., 2011; Shabani et al., 2011, 2012). However, LG/J and SM/J harbor different alleles than the founder strains of these studies (C57BL/6J and DBA/2J) and thus, the most relevant alleles for MA-CPP may be present in other inbred strains. It should also be noted that a phenotypic difference between strains is not necessary for a trait to be heritable (e.g., D1 ACT and D8 ACT; **Figure 2B**; **Table 2**), which may in part be explained by transgressive segregation (Rieseberg et al., 1999).

AIL mice segregating LG/J and SM/J alleles showed a significant MA-CPP that was maximal after 1 week of training on D8 (**Figure 2E**). Thus, we have established a short-term CPP protocol that could be useful in forward genetic studies of MA reward. Because AIL mice did not show any further increase in preference on D15, we focused on the correlation between variables measured from D1 to D8. Importantly, MA-CPP did not correlate with any measure of locomotor activity (**Table 1**), indicating that MA reward is not confounded by test session activity and providing support that it is genetically separable from MA-ACT in this population. This is important because it suggests that the genetic architecture of MA-CPP will be, at least in part, distinct from MA-ACT (Cheng et al., 2010).

We were drastically underpowered to accurately estimate heritability in this study as indicated by our large standard errors of the estimate (SE; **Table 2**). Assuming equal offspring per family (*N* = 2) and random mating, a minimum of 100 midparent-offspring observations were estimated to be required to demonstrate that a trait with a heritability of 0.2 is significantly different from 0 (Klein et al., 1973). Under the same conditions and with an alpha level of 0.05, 60% power is achieved – 400 families would be required to achieve greater than 95% power (Klein, 1974). With this in mind, our heritability estimates for both MA-CPP and MA-ACT using 10 midparent-offspring observations were low (*h*^2^ = 0–0.21, 0–0.1, respectively) and variable (SE = 0–0.33, 0.13–0.31, respectively; **Table 2**). Despite the low heritability estimate for MA-ACT, we previously mapped several QTLs for this phenotype in the F_34_ generation of AIL mice (Cheng et al., 2010). In contrast to MA-ACT, drug-free locomotor activity showed little or no significant difference between parental strains (**Figures 2B,D**); however, it was the most heritable set of traits across both methods of estimation, ranging from 0.33 to 0.68. This observation is consistent with prior reports (Crabbe et al., 1999; Mhyre et al., 2005; Miller et al., 2010; Philip et al., 2010). To summarize, we cannot conclude based on the present set of data whether MA-CPP is sufficiently heritable for QTL mapping.

The heritability of cocaine-CPP was recently estimated to be 0.11 (Philip et al., 2010), which is within our range for MA-CPP (0–0.21;Table 2). In general, heritability studies arenot comparable across different populations and environmental conditions (Visscher et al., 2008). Philip et al. (2010) used a panel of BXD recombinant inbred strains (derived from C57BL/6J and DBA/2J strains) whereas our study examined alleles derived from the LG/J and SM/J strains. Furthermore, different drugs were used (cocaine versus MA) with different time courses of action and the doses may not have been comparable in potency (10 mg/kg cocaine versus 2 mg/kg MA). The CPP procedures were also very different between Philip et al. (2010) versus the present study including (1) the length of training and testing trials (20 min versus 30 min), (2) the number of trials per day (SAL and cocaine trials on the same day versus MA and SAL trials on separate days), (3) the time separating drug and SAL exposures (cocaine trials immediately following SAL trials on the same day versus 24 h separating MA and SAL trials), (4) the total number of drug and SAL trials (six versus eight or 16 trials), (5) the number of chambers within the CPP apparatus (three chambers versus two chambers), and (6) the size of the training chambers (18 cm × 14 cm versus 37.5 cm × 18.75 cm). Importantly, we observed a relatively large increase in time spent on the drug-paired side (100–250 s; **Figures 2A,E**) compared to Philip et al. (2010;25-30 s maximal preference).

Support for MA-CPP as a heritable trait comes from studies where C57BL/6J × DBA/2J F_2_ mice were selected for high and low oral liquid consumption of MA. The amount of MA consumed is thought to measure the motivational properties of MA; this assumption was supported by the high and low lines differing both in MA-CPP (greater in the high line) and MA-conditioned place aversion (CPA; greater in the low line; Wheeler et al., 2009; Shabani et al., 2011). Because MA oral consumption was heritable (*h*^2^ = 0.34–0.35; Wheeler et al., 2009; Shabani et al., 2011) and because MA-CPP showed a correlated response to selection, this suggests that MA-CPP is also heritable. The CPP assay is both complementary to and distinct from the oral consumption paradigm in that it requires mice both to discriminate contexts and to associate a particular context with the motivational effects of the drug.Drug-associated cues induce powerful neurobiological and psychological states in the brain that motivate drug seeking behavior in addicts (Volkow et al., 2011). Thus, identifying the genetic basis of MA-CPP may reveal genes important for both the motivational properties as well as those important for associative learning and cue-associated craving.

Because we are interested both in identifying QTLs for MA-CPP as well as further narrowing the QTLs already identified for MA-ACT (Cheng et al., 2010), the use of the MA-CPP protocol should permit the latter ancillary goal in the more highly recombinant AIL generations. In support, B6 congenic mice capturing a major QTL on chromosome 11 for MA-ACT in the open field (Bryant et al., 2009) captured this same QTL for MA-ACT in the drug-paired chamber of the CPP apparatus (Bryant et al., 2012a), supporting the presumption that the smaller sized CPP chamber may be used to detect the same QTLs for MA-ACT as the open field.

An important limitation of this study was that we only examined a single systemic dose of MA (2 mg/kg) which was based solely on our historical use of this dose in previous QTL studies involving MA-induced locomotor activity. Indeed, lower doses (e.g., 0.5 mg/kg) produced a greater MA-CPP in mice (Shabani et al., 2011). Thus, our findings may be specific to this dose of MA. There are also several other parameters that could potentially change the correlation among CPP and locomotor variables and their heritability estimates (see above). Thus, we have not attempted to completely explore the large and complex parameter space for CPP in the parental strains or in AIL mice.

We have outlined a short-term protocol in AIL mice that may be useful for identifying the genetic basis of the rewarding properties of MA and possibly other drugs of abuse. Our estimates of heritability for MA-CPP and indeed, for MA-ACT were low and underpowered. Because we previously mapped QTLs for MA-ACT in AIL mice, this suggests that we have not accurately estimated the true heritability and thus, it is possible that we will also have success in mapping QTLs for MA-CPP under these conditions. We believe that the minimal extra labor required warrants its use with the hope that multiple MA traits relevant to drug abuse will be mapped. The use of AIL could greatly accelerate the identification of the responsible genes contributing to the conditioned rewarding properties of MA and nicely complements efforts that are currently underway to identify the genetic basis of MA oral consumption (Wheeler et al., 2009). In addition, genes important for the conditioned rewarding properties of drugs of abuse could have important pleiotropic roles in multiple neuropsychiatric conditions affected by the mesolimbic dopaminergic reward pathway including the non-drug addictions, the emotional-affective component of pain and analgesia, Parkinson’s disease, anxiety, and depression.

## Conflict of Interest Statement

The authors declare that the research was conducted in the absence of any commercial or financial relationships that could be construed as a potential conflict of interest.

## References

[B1] Abney M. McPeek M. S. Ober C. (2000). Estimation of variance components of quantitative traits in inbred populations. *Am. J. Hum. Genet.* 66 629–650 10677322 10.1086/302759 PMC1288115

[B2] Bryant C. D. Chang H. P. Zhang J. Wiltshire T. Tarantino L. M. Palmer A. A. (2009). A major QTL on chromosome 11 influences psychostimulant and opioid sensitivity in mice. *Genes Brain Behav.* 8 795–805 19694818 10.1111/j.1601-183X.2009.00525.x PMC3697834

[B3] Bryant C. D. Kole L. A. Guido M. A. Sokoloff G. Palmer A. A. (2012a). Congenic dissection of a major QTL for methamphetamine sensitivity implicates epistasis. *Genes Brain Behav.* 10.1111/j.1601-183X.2012.00795.x [Epub ahead of print]. PMC3691852 22487465

[B4] Bryant C. D. Parker C. C. Zhou L. Olker C. Chandrasekaran R. Y. Wager T. T. Bolivar V. J. Loudon A. S. Vitaterna M. H. Turek F. W. Palmer A. A. (2012b). Csnk1e is a genetic regulator of sensitivity to psychostimulants and opioids. *Neuropsychopharmacology* 37 1026–1035 22089318 10.1038/npp.2011.287 PMC3280656

[B5] Cheng R. Lim J. E. Samocha K. E. Sokoloff G. Abney M. Skol A. D. Palmer A. A. (2010). Genome-wide association studies and the problem of relatedness among advanced intercross lines and other highly recombinant populations. *Genetics* 185 1033–1044 20439773 10.1534/genetics.110.116863 PMC2907190

[B6] Cheverud J. M. Routman E. J. Duarte F. A. van Swinderen B. Cothran K. Perel C. (1996). Quantitative trait loci for murine growth. *Genetics* 142 1305–1319 8846907 10.1093/genetics/142.4.1305 PMC1207127

[B7] Childs E de Wit H. (2009). Amphetamine-induced place preference in humans. *Biol. Psychiatry* 65 900–904 19111278 10.1016/j.biopsych.2008.11.016 PMC2693956

[B8] Childs E de Wit H. (2011). Contextual conditioning enhances the psychostimulant and incentive properties of d-amphetamine in humans. *Addict. Biol.* 10.1111/j.1369-1600.2011.00416.x [Epub ahead of print]. PMC4242554 22129527

[B9] Crabbe J. C. Jarvik L. F. Liston E. H. Jenden D. J. (1983). Behavioral responses to amphetamines in identical twins. *Acta Genet. Med. Gemellol.* 32 139–149 6685963 10.1017/s0001566000006425

[B10] Crabbe J. C. Wahlsten D. Dudek B. C. (1999). Genetics of mouse behavior: interactions with laboratory environment. *Science* 284 1670–1672 10356397 10.1126/science.284.5420.1670

[B11] Darvasi A. Soller M. (1995). Advanced intercross lines, an experimental population for fine genetic mapping. *Genetics* 141 1199–1207 8582624 10.1093/genetics/141.3.1199 PMC1206841

[B12] Di Chiara G. Imperato A. (1988). Drugs abused by humans preferentially increase synaptic dopamine concentrations in the mesolimbic system of freely moving rats. *Proc. Natl. Acad. Sci. U.S.A.* 85 5274–5278 2899326 10.1073/pnas.85.14.5274 PMC281732

[B13] Fergusson D. M. Horwood L. J. Lynskey M. T. Madden P. A. (2003). Early reactions to cannabis predict later dependence. *Arch. Gen. Psychiatry* 60 1033–1039 14557149 10.1001/archpsyc.60.10.1033

[B14] Goldman D. Oroszi G. Ducci F. (2005). The genetics of addictions: uncovering the genes. *Nat. Rev. Genet.* 6 521–532 15995696 10.1038/nrg1635

[B15] Haertzen C. A. Kocher T. R. Miyasato K. (1983). Reinforcements from the first drug experience can predict later drug habits and/or addiction: results with coffee, cigarettes, alcohol, barbiturates, minor and major tranquilizers, stimulants, marijuana, hallucinogens, heroin, opiates and cocaine. *Drug Alcohol Depend.* 11 147–165 6134605 10.1016/0376-8716(83)90076-5

[B16] Hart A. B. de Wit H. Palmer A. A. (2012). Genetic factors modulating the response to stimulant drugs in humans. *Curr. Top. Behav. Neurosci.* 10.1007/7854_2011_187 [Epub ahead of print]. PMC3388157 22261702

[B17] Ho M. K. Goldman D. Heinz A. Kaprio J. Kreek M. J. Li M. D. Munafo M. R. Tyndale R. F. (2010). Breaking barriers in the genomics and pharmacogenetics of drug addiction. *Clin. Pharmacol. Ther.* 88 779–791 20981002 10.1038/clpt.2010.175 PMC3738009

[B18] Klein T. W. (1974). Heritability and genetic correlation: statistical power, population comparisons, and sample size. *Behav. Genet.* 4 171–189 4842094 10.1007/BF01065758

[B19] Klein T. W. DeFries J. C. Finkbeiner C. T. (1973). Heritability and genetic correlation: standard errors of estimates and sample size. *Behav. Genet.* 3 355–364 4776432 10.1007/BF01070218

[B20] Levran O. Londono D. O’Hara K. Nielsen D. A. Peles E. Rotrosen J. Casadonte P. Linzy S. Randesi M. Ott J. Adelson M. Kreek M. J. (2008). Genetic susceptibility to heroin addiction: a candidate-gene association study. *Genes Brain Behav.* 7 720–729 18518925 10.1111/j.1601-183X.2008.00410.x PMC2885890

[B21] Mhyre T. R. Chesler E. J. Thiruchelvam M. Lungu C. Cory-Slechta D. A. Fry J. D. Richfield E. K. (2005). Heritability, correlations and in silico mapping of locomotor behavior and neurochemistry in inbred strains of mice. *Genes Brain Behav.* 4 209–228 15924554 10.1111/j.1601-183X.2004.00102.x

[B22] Miller B. H. Schultz L. E. Gulati A. Su A. I. Pletcher M. T. (2010). Phenotypic characterization of a genetically diverse panel of mice for behavioral despair and anxiety. *PLoS ONE* 5 e14458 10.1371/journal.pone.0014458 PMC3012073 21206921

[B23] Nurnberger J. I. Jr. Gershon E. S. Simmons S. Ebert M. Kessler L. R. Dibble E. D. Jimerson S. S. Brown G. M. Gold P. Jimerson D. C. Guroff J. J. Storch F. I. (1982). Behavioral, biochemical and neuroendocrine responses to amphetamine in normal twins and ‘well-state’ bipolar patients. *Psychoneuroendocrinology* 7 163–176 6891082 10.1016/0306-4530(82)90009-9

[B24] Palmer A. A de Wit H. (2012). Translational genetic approaches to substance use disorders: bridging the gap between mice and humans. *Hum. Genet.* 131 931–939 22170288 10.1007/s00439-011-1123-5 PMC3352994

[B25] Palmer A. A. Verbitsky M. Suresh R. Kamens H. M. Reed C. L. Li N. Burkhart-Kasch S. McKinnon C. S. Belknap J. K. Gilliam T. C. Phillips T. J. (2005). Gene expression differences in mice divergently selected for methamphetamine sensitivity. *Mamm. Genome* 16 291–305 16104378 10.1007/s00335-004-2451-8

[B26] Parker C. C. Cheng R. Sokoloff G. Palmer A. A. (2012). Genome-wide association for methamphetamine sensitivity in an advanced intercross mouse line. *Genes Brain Behav.* 11 52–61 22032291 10.1111/j.1601-183X.2011.00747.x PMC3368015

[B27] Philip V. M. Duvvuru S. Gomero B. Ansah T. A. Blaha C. D. Cook M. N. Hamre K. M. Lariviere W. R. Matthews D. B. Mittleman G. Goldowitz D. Chesler E. J. (2010). High-throughput behavioral phenotyping in the expanded panel of BXD recombinant inbred strains. *Genes Brain Behav.* 9 129–159 19958391 10.1111/j.1601-183X.2009.00540.x PMC2855868

[B28] Phillips T. J. Kamens H. M. Wheeler J. M. (2008). Behavioral genetic contributions to the study of addiction-related amphetamine effects. *Neurosci. Biobehav. Rev.* 32 707–759 18207241 10.1016/j.neubiorev.2007.10.008 PMC2360482

[B29] Rieseberg L. H. Archer M. A. Wayne R. K. (1999). Transgressive segregation, adaptation and speciation. *Heredity (Edinb.)* 83(Pt 4) 363–372 10.1038/sj.hdy.6886170 10583537

[B30] Schuckit M. A. Smith T. L. (2001). The clinical course of alcohol dependence associated with a low level of response to alcohol. *Addiction* 96 903–910 11399221 10.1046/j.1360-0443.2001.96690311.x

[B31] Scibelli A. C. McKinnon C. S. Reed C. Burkhart-Kasch S. Li N. Baba H. Wheeler J. M. Phillips T. J. (2011). Selective breeding for magnitude of methamphetamine-induced sensitization alters methamphetamine consumption. *Psychopharmacology (Berl.)* 214 791–804 21088960 10.1007/s00213-010-2086-2 PMC3320759

[B32] Shabani S. Dobbs L. K. Ford M. M. Mark G. P. Finn D. A. Phillips T. J. (2012). A genetic animal model of differential sensitivity to methamphetamine reinforcement. *Neuropharmacology* 62 2169–2177 22280875 10.1016/j.neuropharm.2012.01.002 PMC3320769

[B33] Shabani S. McKinnon C. S. Reed C. Cunningham C. L. Phillips T. J. (2011). Sensitivity to rewarding or aversive effects of methamphetamine determines methamphetamine intake. *Genes Brain Behav.* 10 625–636 21554535 10.1111/j.1601-183X.2011.00700.x PMC3320762

[B34] Shao J Wu C. F. J. (1989). A general theory for jackknife variance estimation. *Ann. Stat.* 17 1176–1197

[B35] Tzschentke T. M. (2007). Measuring reward with the conditioned place preference (CPP) paradigm: update of the last decade. *Addict. Biol.* 12 227–462 17678505 10.1111/j.1369-1600.2007.00070.x

[B36] Veenstra-VanderWeele J. Qaadir A. Palmer A. A. Cook E. H. Jr. de Wit H. (2006). Association between the casein kinase 1 epsilon gene region and subjective response to D-amphetamine. *Neuropsychopharmacology* 31 1056–1063 16237383 10.1038/sj.npp.1300936

[B37] Visscher P. M. Hill W. G. Wray N. R. (2008). Heritability in the genomics era – concepts and misconceptions. *Nat. Rev. Genet.* 9 255–266 18319743 10.1038/nrg2322

[B38] Volkow N. D. Wang G. J. Fowler J. S. Tomasi D. Telang F. (2011). Addiction: beyond dopamine reward circuitry. *Proc. Natl. Acad. Sci. U.S.A.* 108 15037–15042 21402948 10.1073/pnas.1010654108 PMC3174598

[B39] Wheeler J. M. Reed C. Burkhart-Kasch S. Li N. Cunningham C. L. Janowsky A. Franken F. H. Wiren K. M. Hashimoto J. G. Scibelli A. C. Phillips T. J. (2009). Genetically correlated effects of selective breeding for high and low methamphetamine consumption. *Genes Brain Behav.* 8 758–771 19689456 10.1111/j.1601-183X.2009.00522.x PMC2783502

[B40] Wise R. A. Bozarth M. A. (1987). A psychomotor stimulant theory of addiction. *Psychol. Rev.* 94 469–492 3317472

